# Characteristics of Governmental Public Health Nurses With Recommendations for Public Health Nurse Workforce Planning

**DOI:** 10.1111/phn.13576

**Published:** 2025-06-08

**Authors:** Susan J. Zahner, Katie Gillespie, Kristin Merss, Paula Bizot

**Affiliations:** ^1^ University of Wisconsin‐Madison School of Nursing Madison Wisconsin USA

**Keywords:** COVID‐19 pandemic, nursing education, nurse workforce, public health nurse, public health workforce

## Abstract

**Objectives:**

Characteristics and perceived impact of the COVID‐19 pandemic on the health of governmental public health nurses were compared to all registered nurses.

**Design:**

Cross‐sectional descriptive design using data from the *2022 Wisconsin RN Workforce Survey*.

**Sample:**

The sample included 87,100 registered nurses, including 1350 governmental public health nurses.

**Measurements:**

The online survey was administered during February 2022. Responses to “Working as a nurse” and “Primary place of work” were used to classify governmental public health nurses. Data elements included demographics, employment, income, education, specialized knowledge, certifications, and COVID‐19 pandemic measures.

**Results:**

Compared to all registered nurses, governmental public health nurses reported older age, greater race/ethnic diversity, and higher frequency of the baccalaureate as the highest degree. Most governmental public health nurses reported no plans for additional education. Income for governmental public health nurses lagged income for all registered nurses. The proportion of governmental public health nurses intending to remain in their present type of work for fewer than 10 years was higher than for all registered nurses, as was the proportion of governmental public health nurses who rated their overall health as “worse or much worse” than before the pandemic.

**Conclusion:**

This analysis raises concerns about the size and preparation of the governmental public health nurse workforce given ongoing population health disparities and future health threats.

## 1 Introduction

In the wake of the COVID‐19 pandemic, the United States (US) federal government has been making historic investments in governmental health departments and public health infrastructure across the country. This investment is long overdue as the governmental public health system, including the workforce, has been declining for well over a decade due to persistent underfunding (Alfonso et al. 2021; McKillop and Ilakkuvan 2019; McKillop and Lieberman 2023). The Centers for Disease Control and Prevention (CDC) expects to award more than $4.5 billion over the 5‐year Public Health Infrastructure Grant period, from 2022 to 2027, to “ensure that every U.S. community has the people, services, and systems needed to promote and protect health” (CDC 2023). The three strategic areas included in the grant are workforce, foundational capabilities, and data modernization. The largest portion of the grant funding, $3 billion, is directed toward workforce strategy to help health departments recruit, retain, support, and train the public health workforce (CDC 2022).

The US governmental public health workforce includes individuals who work in federal, state, and local public health agencies. The public health workforce, particularly at state and local levels, has experienced significant turnover following the onset of the COVID‐19 pandemic (de Beaumont Foundation 2022; Leider, Castrucci, et al. 2023). Even before the pandemic, the size of the state and local health department workforce in the United States was shrinking, with significant losses noted between 2008 and 2019 (Leider, Yeager, et al. 2023; Robin and Leep 2017), including a decline of 36% in the numbers of public health nurses (NACCHO 2019). Efforts to define and enumerate the governmental public health workforce have proven challenging, which makes monitoring the trends and clarifying needs for recruitment and retention difficult (Kneipp et al. 2022; Leider, Yeager, et al. 2023). Understanding the characteristics of the current governmental public health workforce is crucial for building a future workforce infrastructure to support and sustain public health services delivery.

Registered nurses (RNs) make up a substantial component of the governmental public health workforce (Beck et al. 2014; Cunningham et al. 2024). As defined by the American Public Health Association, “public health nursing is the practice of promoting and protecting the health of populations using knowledge from nursing, social, and public health sciences” (APHA 2013). This broad definition includes nurses who work in governmental public health settings as well as nurses who work in schools, parishes, and some community‐based nonprofit organizations. In this article, we focus only on RNs working in governmental public health settings at federal, state, and local levels and henceforth refer to the group as governmental public health nurses (GPHNs).

The critical importance of GPHNs working to maintain the public's health and wellness has long been discussed, though the evidence base for GPHN impact is still limited. Research has demonstrated the positive effects of GPHN practice on a variety of health outcomes, such as the relationship of GPHN in executive leadership roles and mortality (Kett et al. 2022), the impact of sustained nurse home visiting on child and maternal outcomes (Molloy et al. 2021), and on improving client knowledge, behavior, and health status across a variety of problems (Huling et al. 2024). The COVID‐19 pandemic exposed limitations of the public health system related to preparedness, surveillance, and capabilities, and highlighted the importance of GPHNs working with communities and between medicine and public health (Bekemeier et al. 2021; Brownson et al. 2020; Guilamo‐Ramos et al. 2021; Kett et al. 2024). The COVID‐19 pandemic placed extreme demands on nurses and was associated with worsening physical and mental health, including increased anxiety, depression, post‐traumatic stress disorder, insomnia, poor diet, and overall increased stress due to working conditions (Kelley et al. 2022; Gwon et al. 2023; Pfender et al. 2022). Information about the pandemic's impact specific to the health of GPHNs is limited, and understanding that impact is important to help retain GPHNs and to improve emergency preparedness planning for future pandemics. Overall, persistent challenging health problems and the constant threat of public health crises point to ongoing needs for a strong GPHN workforce.

Public health infrastructure planning requires information about the workforce specific to individual states and to the characteristics of segments of the workforce, including GPHNs. This information could also be helpful to nursing educators as they plan for curricula revisions and enhancements in baccalaureate and advanced level community and public health nursing programs. This study was undertaken to describe characteristics of the GPHN in the state of Wisconsin. Public health in Wisconsin is a shared responsibility between state and local governments. The state's 85 local health departments are governed by state statutes and administrative rules that define minimum services and staffing requirements (Qualifications for Public Health Professionals 2011; Local Health Officials 2023; Required Services of Local Health Departments 2019) and are required to employ at minimum one licensed, bachelor‐prepared RN.

The Wisconsin state statutes (Nursing Workforce Survey and Grant 2009/2011) require RNs to complete a survey when renewing their professional licenses on a biennial basis. This survey, referred to as the *Wisconsin RN Workforce Survey*, includes responses from all RNs who are licensed to work in the state and features a breadth of questions that is rare among other nursing workforce surveys (Kneipp et al. 2022). The *Wisconsin RN Workforce Survey* provides a data source that allows description and comparison of characteristics of the state's RNs overall and by reported work setting. The 2022 survey included a question about the impact of the COVID‐19 pandemic on perceived overall health (physical and mental), allowing for increased understanding of the impact of the pandemic on GPHNs. In this study, we conducted a secondary analysis of data from the 2022 survey to examine descriptors of GPHNs and to make comparisons to all RNs. GPHNs were defined as RNs employed in positions with local, state, or federal governmental public health agencies in Wisconsin that require an RN license. The specific objectives of our study were to (1) describe the GPHN workforce in Wisconsin, (2) compare demographic characteristics of GPHNs with all RNs living or working in the state, (3) compare the impact of the COVID‐19 pandemic on the overall physical and mental health of GPHNs and all RNs, and (4) provide insights and recommendations to improve the recruitment, retention, support, and training of the GPHN workforce in Wisconsin.

## 2 Methods

This analysis was conducted under an approved data use agreement between the Wisconsin Center for Nursing and the Wisconsin Department of Workforce Development. This study was determined by the University of Wisconsin‐Madison Minimal Risk Research Institutional Review Board to not constitute research with human subjects (IRB# 2023‐0470). The overall survey instrument, administration methods, data management and cleaning methods, including inclusion and exclusion criteria, and results are described in the *Wisconsin 2022 RN Survey Report* (Zahner et al. 2023).

### 2.1 Design and Sample

A secondary analysis using a cross‐sectional descriptive design was conducted. A total of 97,100 RNs completed the *2022 Wisconsin RN Workforce Survey* during the open RN license renewal period in the month of February 2022 (129 were paper mailed‐in surveys, and 96,971 were completed online). After exclusion criteria were applied, a total of 87,100 usable responses from RNs living and working in the state remained in the overall survey dataset (Zahner et al. 2023). The subset of GPHNs was identified using the criteria of current employment status reported as “working as a nurse (i.e., receiving compensation for work requiring licensure or educational preparation as a nurse)” and primary place of work reported as “public health (governmental: federal, state, local).” We compared characteristics of GPHNs to the entire sample of RNs (*n* = 87,100) as described in the full report (Zahner et al. 2023).

### 2.2 Measures

The *2022 Wisconsin RN Workforce Survey* instrument included 82 forced‐choice items and is available in the appendix of the full report (Zahner et al. 2023). The data elements from the survey used for this analysis are shown in Table 1. The total number of responses varied by data element due to missing data. Results are presented as proportions (%) of the total valid responses for each data element, providing the most meaningful descriptive comparison between two groups that are substantially different in size.

**TABLE 1:** Variables and frequencies.

		Proportions of responses (%)	
Variables	Responses	GPHN *N* = 1350	All RN^*^ *N* = 87,100
**Demographic characteristics**	
Race	White	91.9	93.4
	Black/African American	3.9	2.5
	American Indian/Alaska Native	1	0.7
	Asian	2.1	2.6
	Native Hawaiian/Other Pacific Islander	0	0.2
	Other	2.1	1.7
Ethnicity	Hispanic	3.4	2.6
Gender	Woman	93.3	91.6
	Man	6.4	8.1
	Other (nonbinary)	0.3	0.3
Age	Mean (standard deviation)	47.5 (12.9) years	46.1 (13.7) years
**Educational characteristics**			
Highest degree earned in any field	Bachelor's Degree in any field	61.0	50.5
	Graduate (Master's or Doctorate)	14.2	17.4
Highest degree earned in nursing	Associate Degree in Nursing	24.7	30.9
	Bachelor's in Nursing	62.8	50.9
	Graduate (Master's or any Doctorate in nursing)	10.4	15.1
Currently enrolled in nursing education program	Yes	5.2	8.9
Plans for future education	No	75.8	73.9
Advanced practice RN	Yes	4.2	8.8
**Employment characteristics**			
Hours worked/week	Mean (standard deviation)	38.7 (13.6) h	37.4 (12.3) h
Working more hours/week in last year	Yes	34.3	39
Employment setting	Urban	62.2	76.7
	Rural	37.8	23.2
Position change	No change in past year	71.3	71.5
Intention to stay in present type of work	< 10 years	61.3	57.8
**Income**			
Median income for full‐time work	All settings	$70,000	$80,000
Median income by employment setting	Urban	$70,000	$80,000
	Rural	$60,000	$70,000
**Impact of COVID‐19**			
Impact on overall personal health (physical or mental	Better	7.3	8.0
	About the same	39.0	44.2
	Worse	43.3	38.5
	Much worse	10.3	9.3
Covid training	Received training	96.4	88.2
	Training provided by employer	86.8	82.9
**Emergency preparedness training**			
	Received training	75.1	60.8
	Received from employer	69.7	65.3
	Applied training	47.8	22.2

*Source*: 2022 Wisconsin Registered Nurse Survey.

### 2.3 Analytic Strategy

Frequencies or means as appropriate to the data element were generated from the dataset using SPSS for GPHNs and All RNs groups. Differences between the groups were compared.

## 3 Results

Of the 87,100 usable responses included in the dataset from the *2022 Wisconsin RN Workforce Survey*, 1350 (1.5%) were classified as GPHNs.

### 3.1 Demographics

Table 1 displays demographic characteristics of GPHNs and all RNs. Slightly higher proportions of GPHNs (93.3%) reported identifying as women compared to all RNs (91.6%). The mean age for GPHNs (47.5 years) was slightly older than the mean age of all RNs (46.1 years). While a high proportion of RNs identified as White (93.4%), the proportion of GPHNs (91.9%) who so identified was slightly lower. The proportion of GPHNs (3.4%) who identified as Hispanic/Latino/Latinx was higher than the 2.6% reported for all RNs.

### 3.2 Education

A higher proportion of GPHNs hold a bachelor's degree in any field as their highest degree (61%) compared to all RNs (50.5%). However, the proportion of GPHNs that reported having earned graduate degrees (master's or doctoral) was lower at 14.2% compared to that reported by all RNs (17.4%). Specific to degrees in nursing, a higher proportion of GPHNs hold a bachelor of nursing degree as their highest nursing degree (62.8%) compared to all RNs (50.9%). However, the proportion of GPHNs that reported having earned graduate degrees in nursing (master's or doctoral) was lower at 10.4% compared to that reported by all RNs (15.1%). A small percentage (5.2%) of GPHNs reported that they are currently enrolled in an educational program, a proportion lower than that for all RNs (8.9%). The most frequently cited programs in which GPHNs were enrolled were a master's in nursing (2.1%) and a bachelor's in nursing (1.5%). Most GPHNs (75.8%) and RNs (73.9%) reported no plans for additional education. The most cited barriers to further education were the cost of tuition, materials, and books; family or personal reasons; and the cost of lost work time and benefits.

A small proportion of GPHNs (4.2%) reported being advanced practice registered nurses (APRNs) compared to 8.8% of all RNs. Of the APRNs who are GPHNs, 91.2% are credentialed as nurse prescribers. Most APRNs are credentialed as family nurse practitioners overall (55.4%), and among those who work for governmental public health (53.3%).

### 3.3 Employment

On average, GPHNs reported working 38.7 h per week, and all RNs reported working 37.4 h per week. The survey requested information about a change in work hours in the past year (February 2021 to February 2022). The proportion of GPHNs who reported working more hours in the past year was 34.3%, lower than the 39% of all RNs.

A higher proportion of GPHNs (37.8%) work in rural areas (defined by zip code of primary employer) compared to all RNs (23.2%). Most of the GPHNs (71.3%) and all RNs (71.5%) reported no change in either primary position or employer in the year prior to the survey. For those who had made a change, the most cited reason was dissatisfaction with the previous position (17.9% of all RNs; 20.4% of GPHNs), followed by “other” (13.6% of all RNs; 16.7% of GPHNs), promotion or career advancement (13.1% of all RNs; 14.3% of GPHNs), and seeking more convenient hours (9.1% of all RNs; 13.9% of GPHNs). Survey respondents were also asked about their plans to stay in their present type of employment. The majority of GPHNs (61.3%) indicated they intend to remain in their present type of work for less than 10 years, a slightly higher proportion than among all RNs (57.8%).

### 3.4 Emergency Preparedness

The survey asked about emergency preparedness training and the application of that training. A higher proportion (75.1%) of GPHNs compared to all RNs (60.8%) had received such training, and most received the training from their employers (69.7% of GPHNs and 65.3% of all RNs). More GPHNs (47.8%) reported having applied their emergency preparedness training compared to all RNs (22.2%).

### 3.5 Income

Income comparisons were made only for RNs who reported working full‐time (*N* = 57,504). The median income for GPHNs who reported working full‐time was $70,000 per year, lower than the median income for all RNs working full‐time ($80,000). Reported incomes were higher for RNs working in urban locations compared to rural locations. The median income for all RNs working in rural areas was $70,000, while in urban areas it was $80,000. This difference in income by geography held true for GPHNs, where the median income in rural areas was $60,000 and in urban areas was $70,000.

### 3.6 Impact of COVID‐19

**FIGURE 1:**
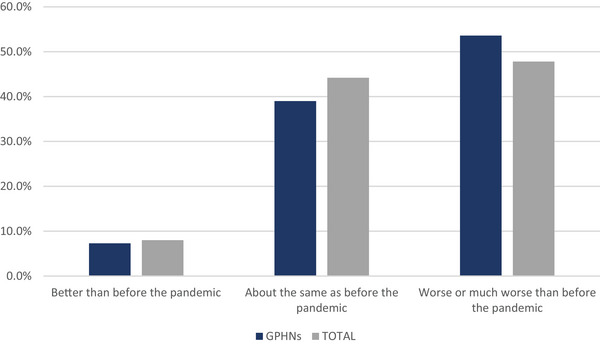
Impact of COVID‐19 pandemic on health. [Colour figure can be viewed at wileyonlinelibrary.com]

The survey asked RNs to rate the impact of the COVID‐19 pandemic on their overall personal health (physical or mental). As shown in Figure 1, 53.6% of GPHNs rated their current health as worse (43.3%) or much worse (10.3%) than before the pandemic, while 7.3% indicated their health was better than before the pandemic, and 39% indicated it was about the same. The negative impact on overall mental and physical health was also apparent for RNs overall, with 38.5% reporting worse health and 9.3% reporting much worse health, while 8% reported better health, and 44.2% reported about the same. When asked about training related to COVID‐19, 96.4% of GPHN respondents indicated they received training on preventing virus transmission, with 86.8% indicating the training was provided by their employer. For all RNs, 88.2% reported receiving training, with 82.9% reporting the training was provided by their employer.

## 4 Discussion

Our study revealed lower incomes reported by GPHNs compared to RNs overall, a disparity that is worse for those working in rural settings. Lower salaries in public health may contribute to challenges in recruiting and retaining nurses and other professionals to public health positions. The *2021 Public Health Workforce Interests and Needs Survey* (de Beaumont Foundation n.d.) found that 27% of public health employees planned to leave the workforce within the year for reasons other than retirement, and pay was cited as the top reason for leaving.

Our study found the proportion of GPHN (62.8%) with bachelor degrees as their highest nursing degree was higher than all RNs (50.9%) in Wisconsin and in a national sample of RNs (50.4%) (Choi et al. 2023). This finding is likely due at least in part to the statutory requirement for local health departments to employ at a minimum one bachelor ‐prepared RN (Qualifications of Public Health Professionals ‐ DHS 139 2011). However, the proportion of RNs with bachelor's degrees in Wisconsin is still well under the recommendation of 80% (Institute of Medicine 2011). Beyond the statutory mandate, baccalaureate nursing education is necessary for GPHN practice given the higher level of emphasis on disease prevention, health promotion, and population health compared to the associate degree level programs. Nursing education for public health is guided by ANA's Public Health Nursing Scope and Standards of Practice (ANA 2022) and *The Essentials: Core Competencies for Professional Nursing Education*, which emphasize these areas of competency required for public health practice (AACN 2021). In Wisconsin and across the country, academic nursing programs are actively revising curricula to accommodate these new essentials, which may result in improving preparation for public health practice among future graduates.

The number and proportion of RNs in Wisconsin who were categorized in prior reports as working in “community health and public health” has declined over the past decade (Department of Health Services, Division of Public Health Informatics 2013; Zahner et al. 2023). This decline mirrors the national overall decrease in the size of the public health workforce between 2008 and 2019 (Cunningham et al. 2024). A lack of adequate numbers of GPHNs may have contributed to limitations in the ability of local public health departments to fully support the public's needs during the COVID‐19 pandemic (Stone et al. 2021; Pittman and Park 2021). The GPHN workforce in Wisconsin is also less diverse by race, ethnicity, and gender than the state's population, which is 49.9% female, 86.4% White (79.5% White alone, not Hispanic), 6.6% Black, 3.3% Asian, 1.2% AI/AN, and 8.1% Hispanic (U.S. Census Bureau 2024). This diversity gap may contribute to ongoing health disparities in Wisconsin, as evidence suggests that racial and ethnic concordance between providers and clients can improve health outcomes (Baker et al. 2024; Jetty et al. 2022). The higher mean age of GPHNs also points to ongoing concerns about replacing the number of GPHNs who will be leaving the workforce due to retirement. The need to increase the number of GPHNs in Wisconsin was recognized in a recent supply and demand forecast, which projected a need for 5.3% more public health nurses (PHN) in the state in 2025 as compared to 2022 (Walsh and del Pilar Casal 2024).

The *2022 Wisconsin RN Workforce Survey* results revealed threats to preparing future nurses for public health practice. Overall, 1169 nurse faculty were identified in the survey, and only 120 (10.3%) of them reported having specialized knowledge and/or experience of 2 or more years in public health (Zahner et al. 2023). Survey results also highlighted a lack of interest on the part of GPHNs in furthering their education to support gaining knowledge and skills for teaching. Less than 20% of GPHNs reported plans to pursue future education in the near term (2 years or less), and 75.8% had no plans for more education. This level of disinterest in nursing education is concerning given the great need to educate future generations of GPHNs.

The relatively low proportion of GPHNs who reported holding graduate degrees was also puzzling given the complexity and scope of specialty GPHN practice, opportunities for leadership roles in public health agencies, and the shortage of faculty in nursing education. The lack of investment in graduate academic education programs focused on public health and population health nursing over recent decades has limited opportunities for nurses to obtain the advanced education required for leadership and faculty roles in public health nursing (Canales and Drevdahl 2014; Bekemeier et al. 2021).

Our understanding of the impact of the COVID‐19 pandemic on GPHNs is limited. In this survey, GPHNs reported a decline in their overall physical and mental health compared to before the COVID‐19 pandemic. Similarly, the national‐level 2021 *Public Health Workforce Interests and Needs Survey* (de Beaumont Foundation and Association of State and Territorial Health Officials 2022) found one in five public health employees said their mental health was either fair or poor. Stress and the impact of the pandemic were even more significant for public health workers in rural areas where access to healthcare is less and isolation is worse (Kett et al. 2023). Rural communities have a greater reliance on public health nurses yet have fewer resources to support their training and retention. As noted by Gwon et al. (2023), in Wisconsin, GPHNs experienced mental and physical exhaustion and a sense of abandoning important work on which their communities rely. These experiences may have been related to GPHNs not working to their full scope of practice during the pandemic as health departments moved them into narrow roles under the Incident Command System structure (Gwon et al. 2023). Our finding that most GPHN were trained in emergency preparedness but after 2 years of pandemic response most reported that they had not applied that training is curious. More research on the roles and lived experiences of GPHNs in emergency response situations is warranted.

### 4.1 Recommendations

Our findings support other reports in the literature that point to the need to improve recruitment, training, retention, and support of GPHNs and to improve data collection processes and access to data for research and planning related to GPHNs (NACNEP 2023; Sumpter et al. 2022).

#### 4.1.1 Recruitment

Reinvestment in the governmental public health infrastructure at all levels, including increasing the overall number of GPHN positions, is vital for improving health outcomes in communities (NACNEP 2023). New graduate nurses and experienced RNs with experience in population‐focused practice, such as those working in schools or other community population‐based settings, could be recruited for governmental public health positions. Incentives such as school loan forgiveness programs, especially in underserved and critical need areas, and competitive salaries could support recruitment of nurses of all backgrounds to the GPHN workforce (NACNEP 2023). Organizations should review their hiring practices, including position descriptions and interview questions, to identify potential barriers that impede the recruitment and inclusion of new graduate nurses and RNs from diverse backgrounds (Coronado et al. 2020; Travis 2023).

#### 4.1.2 Education

Exposing more student nurses to governmental public health work during their nursing education can encourage careers in public health (Zahner and Henriques 2013). Experiences in governmental public health settings can spark curiosity, counter misperceptions, and encourage future nurses to consider public health work. Nursing schools and colleges should collaborate with governmental public health organizations and other population focused community‐based organizations, such as K‐12 schools, to ensure that undergraduate and graduate students have opportunities to learn the public health knowledge, skills, and perspectives required for working in public health settings. Formalized academic‐practice partnerships, including Academic Health Departments, could be useful to share resources and facilitate collaborative strategies to educate student nurses for positions in public health.

Nursing programs should strive to employ faculty with specialization in public health to help enhance student experiences through sharing their knowledge, expertise, and enthusiasm for public health practice. Scholarships, loan forgiveness, shared appointments with public health agencies, and named public health professorships could support recruitment of qualified public health faculty. A greater commitment to funding and expanding doctoral programs (Doctor of Nursing Practice and PhD) in Advanced Public Health Nursing and other graduate level public health education programs, including residencies and advanced fellowships in leadership, would help expand the number of faculty prepared to educate the future public health nurse workforce (Bekemeier et al. 2021; NACNEP 2023). Additional investments in education programs to train more nurses for public health nursing practice, education, and leadership roles are needed along with targeted incentives to address both perceived and actual barriers to higher education (Bekemeier et al. 2021; NACNEP 2023). A new effort by the National Board of Public Health Examiners to create a new Public Health Nursing Certification program could help encourage GPHNs to incrementally pursue more education and drive preparation for leadership, teaching, and research roles (National Board of Public Health Examiners 2025).

#### 4.1.3 Retention

Nurse residency programs have been successful in supporting new graduate nurses in their transitions to practice, demonstrating outcomes including higher job satisfaction and retention (Letourneau and Fater 2015). The *New to Public Health Residency Program*, developed in Wisconsin through a collaboration between academia and practice, has provided education, mentoring, and support to new public health practice nurses and professionals in the state and beyond (Manske et al. 2022). Making this or similar programs more widely available and more specific in addressing competency development for GPHNs would greatly enhance the transition to public health roles for many RNs.

Higher salaries in public health, particularly in rural areas, could go a long way to easing the financial disincentives associated with working as a GPHN and encourage both recruitment and retention. New mechanisms for funding governmental public health to support higher salaries and more positions are needed, including shifting away from heavy dependence on local property taxes and other revenue sources that are vulnerable to fluctuations of the economy and real estate markets (Reschovsky and Zahner 2013).

#### 4.1.4 Support

The lessons of the COVID‐19 pandemic must not be forgotten. Support for GPHN well‐being is key to mitigating the effects of burnout, reducing turnover, and promoting physical and mental health. Providing access to employee assistance programs, wellness strategies such as exercise, meditation, and mental health services, along with offering flexible schedules, encouraging time‐off to avoid overwork, and planning for additional staffing in times of public health emergency can help support GPHN and other public health professionals (Bryant‐Genevier et al. 2021; Gwon et al. 2023; Martin et al. 2024).

#### 4.1.5 Data Collection and Access

Most RN workforce survey reports, including the *National Sample Survey of Registered Nurses* (US DHHS, Health Resources, and Services Administration 2019), present combined data on nurses working in community and public health with limited information on the nurses’ characteristics (Castner et al. 2023; Choi et al. 2023; Pittman and Park 2021). While the Wisconsin survey, administered biennially since 2010, collects substantial data on nurses’ characteristics and employment settings, access to the data for research purposes has been limited given its statutory purpose to collect data relevant to the state's RNs for workforce policy and planning. Overall, processes for enumerating GPHNs and understanding their demographic, employment, and education characteristics are weak and inconsistent across states. Improvements in data collection processes across the country are needed to improve understanding about RNs in population health roles and to track changes over time (NACNEP 2023). Data collected through state‐based RN workforce surveys could be used more broadly in research, education, and for comparison across states and workplace settings if made more accessible through flexible electronic data portals and efficient processes for obtaining data use agreements. Consideration could be given to informing RNs of the potential uses of their survey responses for research at the time data are collected to assure participant knowledge and consent for using their responses for research purposes.

### 4.2 Limitations

There are limitations to this analysis that should be considered when interpreting the results. The data were from RNs living or working in one state, and thus the findings cannot be directly generalized to other states. The sample used for this analysis does not include all RNs licensed in Wisconsin, because RNs not living or working in the state and newly licensed RNs not yet required to re‐license were excluded from the overall survey report (Zahner et al. 2023). The survey data are cross‐sectional in nature and were collected in February 2022. As with many surveys, there may have been some variation in how respondents interpreted the questions and response options, resulting in possible under‐ or over‐counting of responses. Our approach excluded RNs working in governmental public health settings who did not self‐report working in “nurse” positions. This may have resulted in excluding nurses working in leadership or other positions that were not interpreted by respondents as having a nursing degree requirement, resulting in the undercounting of GPHNs. In addition, the ability to identify differences between groups may have been limited by the differences in group size and the fact that the GPHN group was a subset of the large “all RN” group. Finally, the purpose of the [STATE] *RN Survey* data is for state workforce planning and not research. As a result, we were limited to descriptive analyses and did not test the statistical significance of differences between groups.

## 5 Conclusion

As in other states, Wisconsin routinely collects RN workforce data important for nursing education and nursing workforce policy and planning. While this report compared and contrasted characteristics of GPHN and the overall RN population in one state, the findings are of interest more broadly given the relative lack of data available concerning the public health nurse workforce nationally. This snapshot raises concerns about the size and academic preparation of the public health nursing workforce that should be addressed considering ongoing population health disparities, threats related to climate change, and the potential for future pandemics. Additional investments in public health nursing education programs are needed to ensure a vibrant and successful future public health nurse workforce.

## Ethics Statement

This analysis was conducted under an approved data use agreement between the Wisconsin Center for Nursing and the Wisconsin Department of Workforce Development. This study was determined by the University of Wisconsin‐Madison Minimal Risk Research Institutional Review Board to not constitute research with human subjects.

## Conflicts of Interest

The authors declare no conflicts of interest.

## Data Availability

Some of the data that support the findings of this study are available in a published report (Zahner, S. J., Pinekenstein, B., Henriques, J., Merss, K. B., LeClair, J., Alnuaimi, N., and Krainak, K. [2023]. Wisconsin 2022 RN Workforce Survey Report. Wisconsin Center for Nursing, Inc.) available for download from the Wisconsin Center for Nursing at https://wicenterfornursing.org/rn‐lpn‐survey‐reports/name. Additional data were obtained through a Data Use Agreement between the Wisconsin Center for Nursing and the Wisconsin Department of Workforce Development and are not in the public domain.
